# The genome sequence of the yellow-girdled *Dasysyrphus*, *Dasysyrphus tricinctus *(Fallén, 1817)

**DOI:** 10.12688/wellcomeopenres.22892.1

**Published:** 2024-10-01

**Authors:** Liam M. Crowley, Katie J Woodcock, University of Oxford and Wytham Woods Genome Acquisition Lab, Darwin Tree of Life Barcoding collective, Wellcome Sanger Institute Tree of Life Management, Samples and Laboratory team, Wellcome Sanger Institute Scientific Operations: Sequencing Operations, Wellcome Sanger Institute Tree of Life Core Informatics team, Tree of Life Core Informatics collective, Darwin Tree of Life Consortium

**Affiliations:** 1 University of Oxford, Oxford, England, UK; 2 Wellcome Sanger Institute, Hinxton, England, UK

**Keywords:** Dasysyrphus tricinctus, yellow-girdled Dasysyrphus, genome sequence, chromosomal, Diptera

## Abstract

We present a genome assembly from an individual male *Dasysyrphus tricinctus* (the yellow-girdled *Dasysyrphus*; Arthropoda; Insecta; Diptera; Syrphidae). The genome sequence spans 1,054.90 megabases. Most of the assembly is scaffolded into 5 chromosomal pseudomolecules, including the X sex chromosome. The mitochondrial genome has also been assembled and is 18.39 kilobases in length.

## Species taxonomy

Eukaryota; Opisthokonta; Metazoa; Eumetazoa; Bilateria; Protostomia; Ecdysozoa; Panarthropoda; Arthropoda; Mandibulata; Pancrustacea; Hexapoda; Insecta; Dicondylia; Pterygota; Neoptera; Endopterygota; Diptera; Brachycera; Muscomorpha; Eremoneura; Cyclorrhapha; Aschiza; Syrphoidea; Syrphidae; Syrphinae; Syrphini; *Dasysyrphus*; *Dasysyrphus tricinctus* (Fallén 1817) (NCBI:txid1244581).

## Background

*Dasysyrphus tricinctus* (Fallén 1817), also known as the yellow-girdled *Dasysyrphu*s, is a widespread hoverfly species found across the UK and Ireland ( Ball & Morris, 2015; Stubbs & Falk, 2002).

The species is easily identifiable by its distinctive triple abdominal marking pattern ( Ball & Morris, 2015). The frontmost abdominal markings on tergite 2 are narrow bars or sometimes small oval spots, the hind markings on tergite 4 are thin bars, and the middle markings on tergite 3 are significantly larger and broader bars ( Ball & Morris, 2015; Stubbs & Falk, 2002; van Veen, 2014). *D. tricinctus* can be found from March to October, with two annual peaks in late May to early June and late August to early September in Ireland ( Ball & Morris, 2015; Stubbs & Falk, 2002). The larvae of this species, which have a bark-like, spiky appearance, rest camouflaged on tree branches during the day and feed on aphids among foliage at night. They have been found among aphid-infested sycamore ( *Acer pseudoplatanus*) leaves and observed consuming lepidopterous caterpillars and sawfly larvae ( Ball & Morris, 2000; Ball & Morris, 2015; Rotheray, 1993; Stubbs & Falk, 2002). *D. tricinctus* is a woodland hoverfly species and can commonly be found in broadleaf and coniferous woodlands and heathlands ( Ball & Morris, 2000; Ball & Morris, 2015; Stubbs & Falk, 2002). Adults visit a wide range of floral resources, displaying a preference for yellow flowers such as buttercups and Dandelions ( Ball & Morris, 2000; Ball & Morris, 2015). Generation of a chromosomal-level reference genome for *Dasysyrphus tricinctus* provides a valuable tool to further the understanding of this hoverfly species.

## Genome sequence report

**Figure 1.**
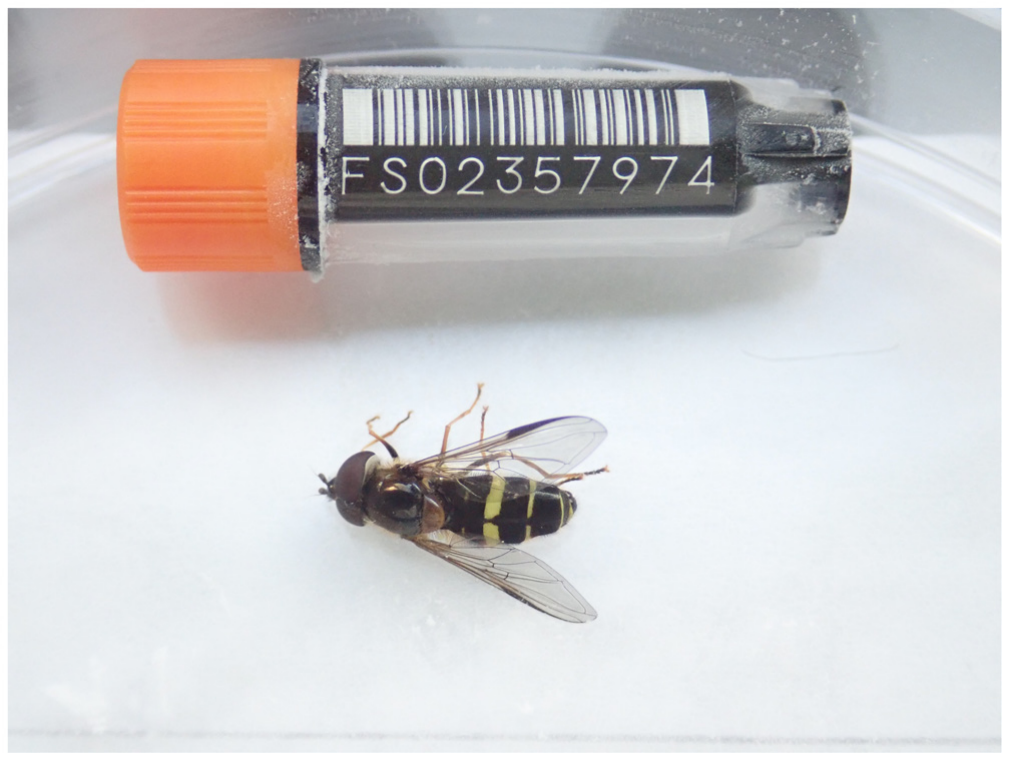
Photograph of the *Dasysyrphus tricinctus* (idDasTrii1) specimen used for genome sequencing.

The genome of an adult male *Dasysyrphus tricinctus* ( Figure 1) was sequenced using Pacific Biosciences single-molecule HiFi long reads, generating a total of 19.01 Gb (gigabases) from 1.28 million reads, providing approximately 17-fold coverage. Primary assembly contigs were scaffolded with chromosome conformation Hi-C data, which produced 139.44 Gbp from 923.44 million reads, yielding an approximate coverage of 132-fold. Specimen and sequencing information is summarised in Table 1.

**Table 1.** Specimen and sequencing data for *Dasysyrphus tricinctus*.

Project information			
**Study title**	Dasysyrphus tricinctus (yellow-girdled Dasysyrphus)		
**Umbrella BioProject**	PRJEB64114		
**Species**	*Dasysyrphus tricinctus*		
**BioSample**	SAMEA7520034		
**NCBI taxonomy ID**	1244581		
Specimen information			
**Technology**	**ToLID**	**BioSample accession**	**Organism part**
**PacBio long read sequencing**	idDasTrii1	SAMEA7520115	head and thorax
**Hi-C sequencing**	idDasTrii1	SAMEA7520116	abdomen
Sequencing information			
**Platform**	**Run accession**	**Read count**	**Base count (Gb)**
**Hi-C HiSeq X Ten**	ERR11679425	9.23e+08	139.44
**PacBio Sequel II**	ERR12040335	1.28e+06	19.01

**Figure 2.**
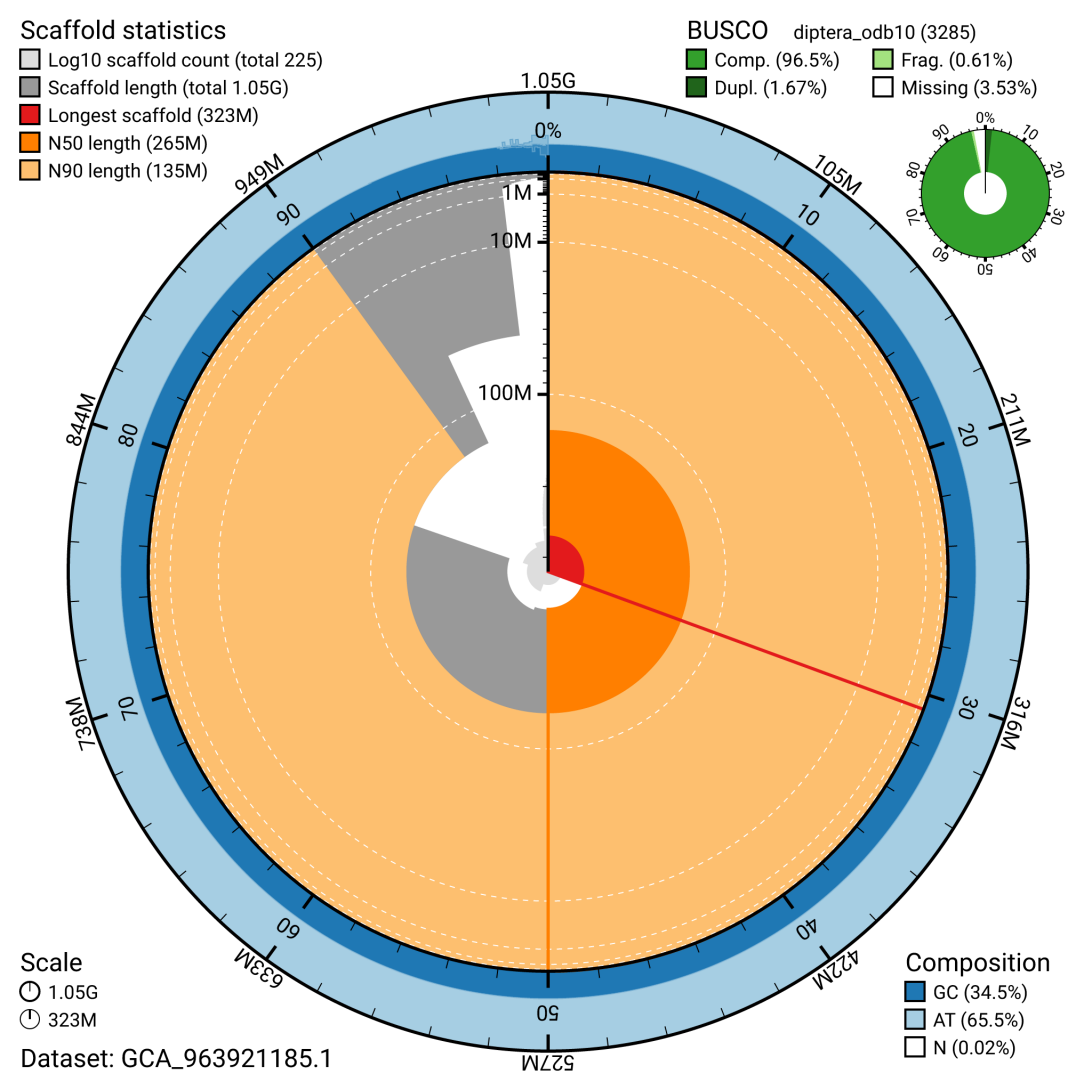
Genome assembly of *Dasysyrphus tricinctus*, idDasTrii1.1: metrics. The BlobToolKit snail plot shows N50 metrics and BUSCO gene completeness. The main plot is divided into 1,000 size-ordered bins around the circumference with each bin representing 0.1% of the 1,054,883,318 bp assembly. The distribution of scaffold lengths is shown in dark grey with the plot radius scaled to the longest scaffold present in the assembly (322,848,792 bp, shown in red). Orange and pale-orange arcs show the N50 and N90 scaffold lengths (264,795,841 and 134,610,944 bp), respectively. The pale grey spiral shows the cumulative scaffold count on a log scale with white scale lines showing successive orders of magnitude. The blue and pale-blue area around the outside of the plot shows the distribution of GC, AT and N percentages in the same bins as the inner plot. A summary of complete, fragmented, duplicated and missing BUSCO genes in the diptera_odb10 set is shown in the top right. An interactive version of this figure is available at https://blobtoolkit.genomehubs.org/view/GCA_963921185.1/dataset/GCA_963921185.1/snail.

**Figure 3.**
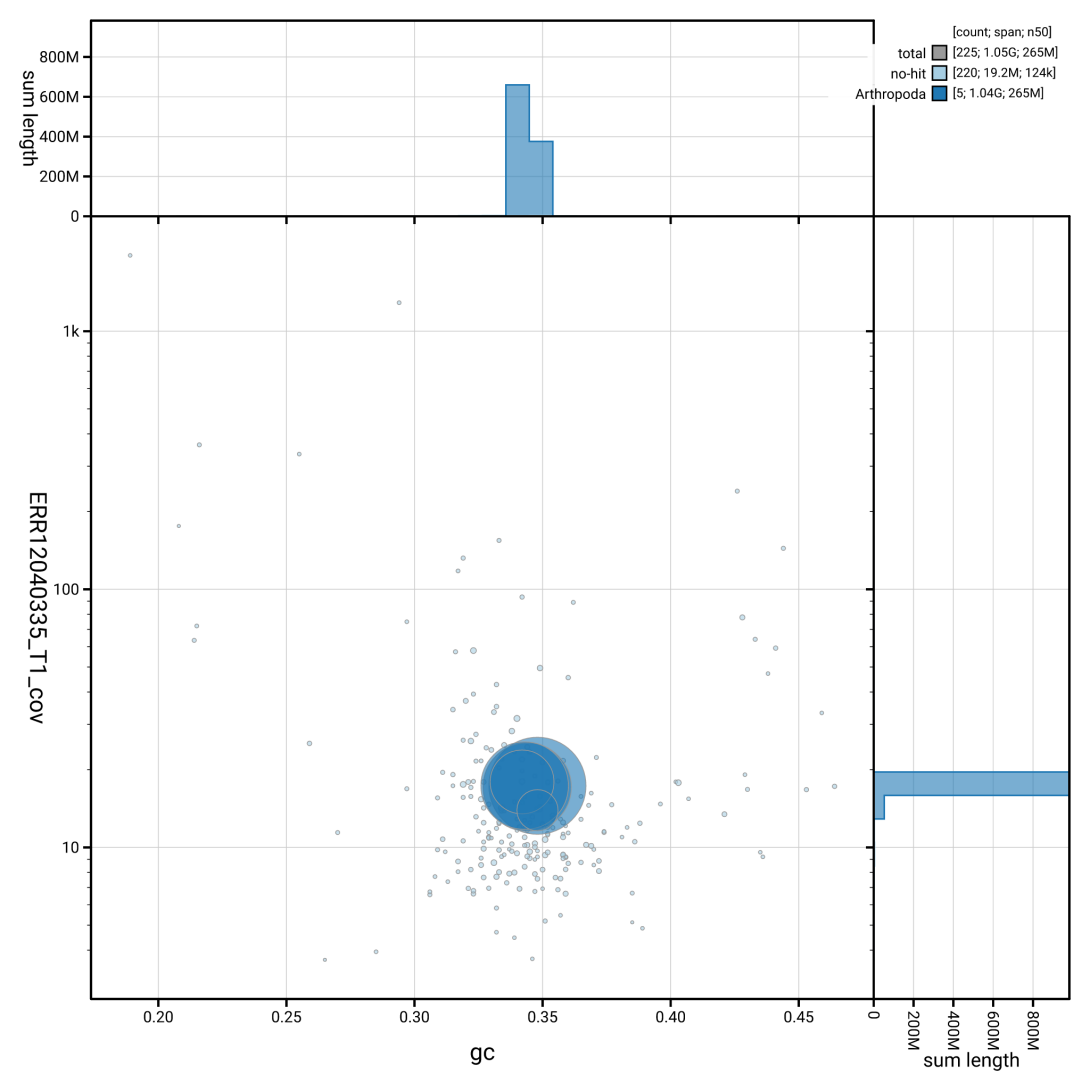
Genome assembly of *Dasysyrphus tricinctus*, idDasTrii1.1: BlobToolKit GC-coverage plot. Blob plot of base coverage in ERR12040335 against GC proportion for sequences in assembly GCA_963921185.1. Sequences are coloured by phylum. Circles are sized in proportion to sequence length. Histograms show the distribution of sequence length sum along each axis. An interactive version of this figure is available at https://blobtoolkit.genomehubs.org/view/GCA_963921185.1/dataset/GCA_963921185.1/blob.

**Figure 4.**
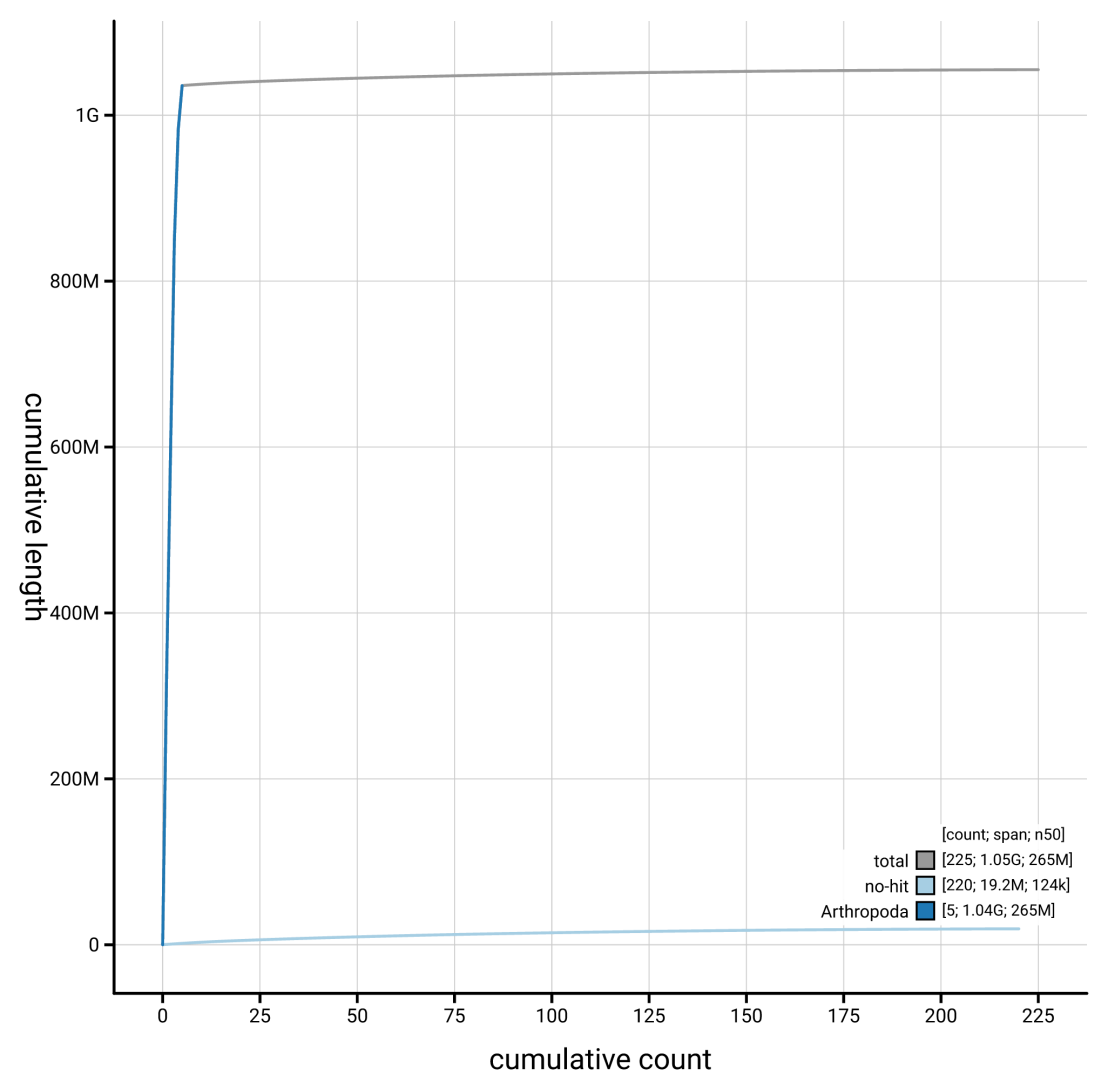
Genome assembly of *Dasysyrphus tricinctus* idDasTrii1.1: BlobToolKit cumulative sequence plot. The grey line shows cumulative length for all sequences. Coloured lines show cumulative lengths of sequences assigned to each phylum using the buscogenes taxrule. An interactive version of this figure is available at https://blobtoolkit.genomehubs.org/view/GCA_963921185.1/dataset/GCA_963921185.1/cumulative.

**Figure 5.**
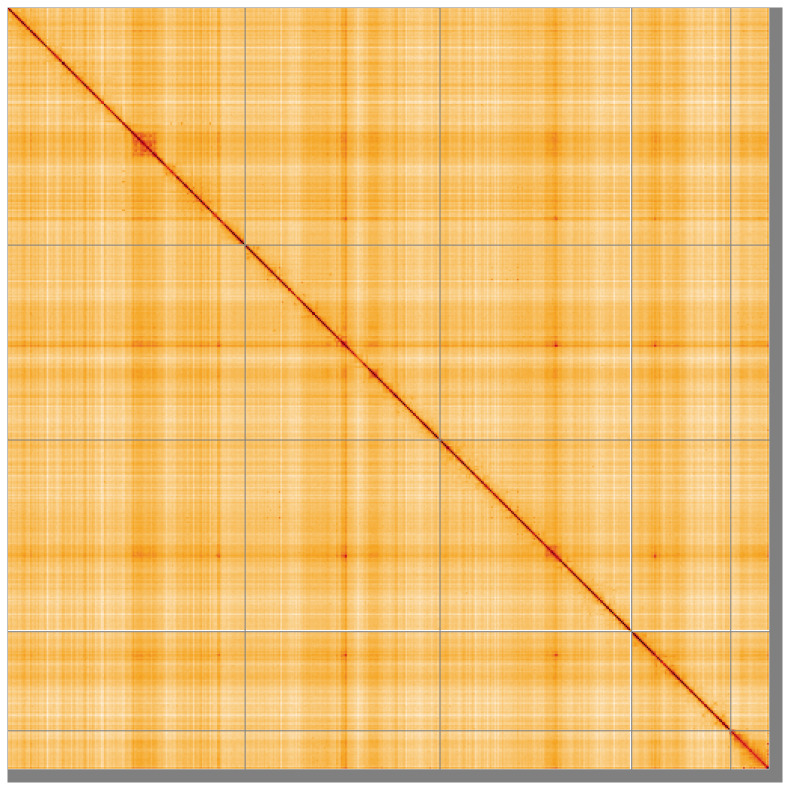
Genome assembly of *Dasysyrphus tricinctus* idDasTrii1.1: Hi-C contact map of the idDasTrii1.1 assembly, visualised using HiGlass. Chromosomes are shown in order of size from left to right and top to bottom. An interactive version of this figure may be viewed at https://genome-note-higlass.tol.sanger.ac.uk/l/?d=Dg7P6jmSTA27DPIEQOvzEg.

Manual assembly curation corrected 253 missing joins or mis-joins and 170 haplotypic duplications, reducing the assembly length by 11.92% and the scaffold number by 35.16%, and decreasing the scaffold N50 by 7.0%. The final assembly has a total length of 1,054.90 Mb in 224 sequence scaffolds, with 831 gaps. The scaffold N50 is 264.8 Mb ( Table 2). The snail plot in Figure 2 provides a summary of the assembly statistics, while the distribution of assembly scaffolds on GC proportion and coverage is shown in Figure 3. The cumulative assembly plot in Figure 4 shows curves for subsets of scaffolds assigned to different phyla. Most (98.17%) of the assembly sequence was assigned to 5 chromosomal-level scaffolds, representing 4 autosomes and the X sex chromosome. Chromosome-scale scaffolds confirmed by the Hi-C data are named in order of size ( Figure 5; Table 3). Chromosome X was assigned based on read coverage statistics. The species appears to be an XO male. While not fully phased, the assembly deposited is of one haplotype. Contigs corresponding to the second haplotype have also been deposited. The mitochondrial genome was also assembled and can be found as a contig within the multifasta file of the genome submission.

**Table 2.** Genome assembly data for *Dasysyrphus tricinctus*, idDasTrii1.1.

Genome assembly		
Assembly name	idDasTrii1.1	
Assembly accession	GCA_963921185.1	
*Accession of alternate haplotype*	*GCA_963921175.1*	
Span (Mb)	1,054.90	
Number of contigs	1,056	
Contig N50 length (Mb)	2.4	
Number of scaffolds	224	
Scaffold N50 length (Mb)	264.8	
Longest scaffold (Mb)	322.85	
Assembly metrics *		*Benchmark*
Consensus quality (QV)	57.1	*≥ 50*
*k*-mer completeness	99.99%	*≥ 95%*
BUSCO **	C:96.3%[S:94.6%,D:1.7%], F:0.7%,M:3.0%,n:3,285	*C ≥ 95%*
Percentage of assembly mapped to chromosomes	98.17%	*≥ 95%*
Sex chromosomes	X	*localised homologous pairs*
Organelles	Mitochondrial genome: 18.39 kb	*complete single alleles*

* Assembly metric benchmarks are adapted from column VGP-2020 of “Table 1: Proposed standards and metrics for defining genome assembly quality” from Rhie *et al.* (2021). ** BUSCO scores based on the diptera_odb10 BUSCO set using version 5.4.3. C = complete [S = single copy, D = duplicated], F = fragmented, M = missing, n = number of orthologues in comparison. A full set of BUSCO scores is available at https://blobtoolkit.genomehubs.org/view/Dasysyrphus_tricinctus/dataset/GCA_963921185.1/busco.

**Table 3.** Chromosomal pseudomolecules in the genome assembly of *Dasysyrphus tricinctus*, idDasTrii1.

INSDC accession	Name	Length (Mb)	GC%
OY992530.1	1	322.85	35.0
OY992531.1	2	264.8	34.5
OY992532.1	3	260.39	34.5
OY992533.1	4	134.61	34.0
OY992534.1	X	53.02	35.0
OY992535.1	MT	0.02	19.0

The estimated Quality Value (QV) of the final assembly is 57.1 with *k*-mer completeness of 99.99%, and the assembly has a BUSCO v5.4.3 completeness of 96.3% (single = 94.6%, duplicated = 1.7%), using the diptera_odb10 reference set ( *n* = 3,285).

Metadata for specimens, BOLD barcode results, spectra estimates, sequencing runs, contaminants and pre-curation assembly statistics are given at https://links.tol.sanger.ac.uk/species/1244581.

## Methods

### Sample acquisition

An adult male *Dasysyrphus tricinctus* (specimen ID Ox000163, ToLID idDasTrii1) was collected from Wytham Woods, Oxfordshire (biological vice-county Berkshire), UK (latitude 51.77, longitude –1.33) on 2019-08-13 by netting. The specimen was collected and identified by Liam Crowley (University of Oxford) and preserved on dry ice.

The initial species identification was verified by an additional DNA barcoding process according to the framework developed by Twyford *et al*. (2024). A small sample was dissected from the specimens and stored in ethanol, while the remaining parts of the specimen were shipped on dry ice to the Wellcome Sanger Institute (WSI). The tissue was lysed, the COI marker region was amplified by PCR, and amplicons were sequenced and compared to the BOLD database, confirming the species identification ( Crowley *et al.*, 2023). Following whole genome sequence generation, the relevant DNA barcode region was also used alongside the initial barcoding data for sample tracking at the WSI ( Twyford *et al.*, 2024). The standard operating procedures for Darwin Tree of Life barcoding have been deposited on protocols.io ( Beasley *et al.*, 2023).

### Nucleic acid extraction

The workflow for high molecular weight (HMW) DNA extraction at the WSI Tree of Life Core Laboratory includes a sequence of core procedures: sample preparation and homogenisation, DNA extraction, fragmentation, and clean-up. Detailed methods developed by the WSI Tree of Life Core Laboratory are publicly available on protocols.io ( Denton *et al.*, 2023b).

The idDasTrii1 sample was weighed and dissected on dry ice ( Jay *et al.*, 2023) and tissue from the head and thorax was homogenised using a PowerMasher II tissue disruptor ( Denton *et al.*, 2023a). HMW DNA was extracted using the Automated MagAttract v1 protocol ( Sheerin *et al.*, 2023). DNA was sheared into an average fragment size of 12–20 kb in a Megaruptor 3 system with speed setting 30 ( Todorovic *et al.*, 2023). Sheared DNA was purified by solid-phase reversible immobilisation, using AMPure PB beads to eliminate shorter fragments and concentrate the DNA ( Strickland *et al.*, 2023). The concentration of the sheared and purified DNA was assessed using a Nanodrop spectrophotometer and Qubit Fluorometer using the Qubit dsDNA High Sensitivity Assay kit. Fragment size distribution was evaluated by running the sample on the FemtoPulse system.

### Sequencing

Pacific Biosciences HiFi circular consensus DNA sequencing libraries were constructed according to the manufacturers’ instructions. DNA sequencing was performed by the Scientific Operations core at the WSI on a Pacific Biosciences Sequel II instrument. Hi-C data were also generated from abdomen tissue of idDasTrii1 using the Arima-HiC v2 kit. The Hi-C sequencing was performed using paired-end sequencing with a read length of 150 bp on the HiSeq X Ten instrument.

### Genome assembly, curation and evaluation

***Assembly***

The PacBio HiFi reads were first assembled using Hifiasm ( Cheng *et al.*, 2021) with the --primary option. Haplotypic duplications were identified and removed using purge_dups ( Guan *et al.*, 2020). The Hi-C reads were mapped to the primary contigs using bwa-mem2 ( Vasimuddin *et al.*, 2019). The contigs were further scaffolded using the provided Hi-C data ( Rao *et al.*, 2014) in YaHS ( Zhou *et al.*, 2023) with the --break option. The scaffolded assemblies were evaluated using Gfastats ( Formenti *et al.*, 2022), BUSCO ( Manni *et al.*, 2021) and MERQURY.FK ( Rhie *et al.*, 2020).

The mitochondrial genome was assembled using MitoHiFi ( Uliano-Silva *et al.*, 2023), which runs MitoFinder ( Allio *et al.*, 2020) and uses these annotations to select the final mitochondrial contig and to ensure the general quality of the sequence.

***Assembly curation***

The assembly was decontaminated using the Assembly Screen for Cobionts and Contaminants (ASCC) pipeline (article in preparation). Flat files and maps used in curation were generated in TreeVal ( Pointon *et al.*, 2023) Manual curation was primarily conducted using PretextView ( Harry, 2022)( Harry, 2022), with additional insights provided by JBrowse2 ( Diesh *et al.*, 2023) and HiGlass ( Kerpedjiev *et al.*, 2018). Scaffolds were visually inspected and corrected as described by Howe *et al*. (2021). Any identified contamination, missed joins, and mis-joins were corrected, and duplicate sequences were tagged and removed. The sex chromosome was identified based on read coverage statistics. The entire process is documented at https://gitlab.com/wtsi-grit/rapid-curation (article in preparation).

***Evaluation of the final assembly***

The final assembly was post-processed and evaluated with the three Nextflow ( Di Tommaso *et al.*, 2017) DSL2 pipelines “sanger-tol/readmapping” ( Surana *et al.*, 2023a)( Surana *et al.*, 2023a), “sanger-tol/genomenote” ( Surana *et al.*, 2023b), and “sanger-tol/blobtoolkit” ( Muffato *et al.*, 2024). The pipeline sanger-tol/readmapping aligns the Hi-C reads with bwa-mem2 ( Vasimuddin *et al.*, 2019) and combines the alignment files with SAMtools ( Danecek *et al.*, 2021). The sanger-tol/genomenote pipeline transforms the Hi-C alignments into a contact map with BEDTools ( Quinlan & Hall, 2010) and the Cooler tool suite ( Abdennur & Mirny, 2020), which is then visualised with HiGlass ( Kerpedjiev *et al.*, 2018). It also provides statistics about the assembly with the NCBI datasets ( Sayers *et al.*, 2024) report, computes *k*-mer completeness and QV consensus quality values with FastK and MERQURY.FK, and a completeness assessment with BUSCO ( Manni *et al.*, 2021).

The sanger-tol/blobtoolkit pipeline is a Nextflow port of the previous Snakemake Blobtoolkit pipeline ( Challis *et al.*, 2020). It aligns the PacBio reads with SAMtools and minimap2 ( Li, 2018) and generates coverage tracks for regions of fixed size. In parallel, it queries the GoaT database ( Challis *et al.*, 2023) to identify all matching BUSCO lineages to run BUSCO ( Manni *et al.*, 2021). For the three domain-level BUSCO lineage, the pipeline aligns the BUSCO genes to the Uniprot Reference Proteomes database ( Bateman *et al.*, 2023) with DIAMOND ( Buchfink *et al.*, 2021) blastp. The genome is also split into chunks according to the density of the BUSCO genes from the closest taxonomically lineage, and each chunk is aligned to the Uniprot Reference Proteomes database with DIAMOND blastx. Genome sequences that have no hit are then chunked with seqtk and aligned to the NT database with blastn ( Altschul *et al.*, 1990). All those outputs are combined with the blobtools suite into a blobdir for visualisation.

The genome assembly and evaluation pipelines were developed using the nf-core tooling ( Ewels *et al.*, 2020), use MultiQC ( Ewels *et al.*, 2016), and make extensive use of the Conda package manager, the Bioconda initiative ( Grüning *et al.*, 2018), the Biocontainers infrastructure ( da Veiga Leprevost *et al.*, 2017), and the Docker ( Merkel, 2014) and Singularity ( Kurtzer *et al.*, 2017) containerisation solutions.

Table 4 contains a list of relevant software tool versions and sources.

**Table 4.** Software tools: versions and sources.

Software tool	Version	Source
BEDTools	2.30.0	https://github.com/arq5x/bedtools2
BLAST	2.14.0	ftp://ftp.ncbi.nlm.nih.gov/blast/executables/blast+/
BlobToolKit	4.3.7	https://github.com/blobtoolkit/blobtoolkit
BUSCO	5.4.3 and 5.5.0	https://gitlab.com/ezlab/busco
bwa-mem2	2.2.1	https://github.com/bwa-mem2/bwa-mem2
Cooler	0.8.11	https://github.com/open2c/cooler
DIAMOND	2.1.8	https://github.com/bbuchfink/diamond
fasta_windows	0.2.4	https://github.com/tolkit/fasta_windows
FastK	427104ea91c78c3b8b8b49f1a7d6bbeaa869ba1c	https://github.com/thegenemyers/FASTK
Gfastats	1.3.6	https://github.com/vgl-hub/gfastats
GoaT CLI	0.2.5	https://github.com/genomehubs/goat-cli
Hifiasm	0.19.8-r603	https://github.com/chhylp123/hifiasm
HiGlass	44086069ee7d4d3f6f3f0012569789ec138f42b84aa44357826c0b6753eb28de	https://github.com/higlass/higlass
Merqury.FK	d00d98157618f4e8d1a9190026b19b471055b22e	https://github.com/thegenemyers/MERQURY.FK
MitoHiFi	3	https://github.com/marcelauliano/MitoHiFi
MultiQC	1.14, 1.17, and 1.18	https://github.com/MultiQC/MultiQC
NCBI Datasets	15.12.0	https://github.com/ncbi/datasets
Nextflow	23.04.0-5857	https://github.com/nextflow-io/nextflow
PretextView	0.2	https://github.com/sanger-tol/PretextView
purge_dups	1.2.5	https://github.com/dfguan/purge_dups
samtools	1.16.1, 1.17, and 1.18	https://github.com/samtools/samtools
sanger-tol/ascc	-	https://github.com/sanger-tol/ascc
sanger-tol/genomenote	1.1.1	https://github.com/sanger-tol/genomenote
sanger-tol/readmapping	1.2.1	https://github.com/sanger-tol/readmapping
Seqtk	1.3	https://github.com/lh3/seqtk
Singularity	3.9.0	https://github.com/sylabs/singularity
TreeVal	1.0.0	https://github.com/sanger-tol/treeval
YaHS	1.2a.2	https://github.com/c-zhou/yahs

### Wellcome Sanger Institute – Legal and Governance

The materials that have contributed to this genome note have been supplied by a Darwin Tree of Life Partner. The submission of materials by a Darwin Tree of Life Partner is subject to the **‘Darwin Tree of Life Project Sampling Code of Practice’**, which can be found in full on the Darwin Tree of Life website here. By agreeing with and signing up to the Sampling Code of Practice, the Darwin Tree of Life Partner agrees they will meet the legal and ethical requirements and standards set out within this document in respect of all samples acquired for, and supplied to, the Darwin Tree of Life Project.

Further, the Wellcome Sanger Institute employs a process whereby due diligence is carried out proportionate to the nature of the materials themselves, and the circumstances under which they have been/are to be collected and provided for use. The purpose of this is to address and mitigate any potential legal and/or ethical implications of receipt and use of the materials as part of the research project, and to ensure that in doing so we align with best practice wherever possible. The overarching areas of consideration are:

- Ethical review of provenance and sourcing of the material
- Legality of collection, transfer and use (national and international)

Each transfer of samples is further undertaken according to a Research Collaboration Agreement or Material Transfer Agreement entered into by the Darwin Tree of Life Partner, Genome Research Limited (operating as the Wellcome Sanger Institute), and in some circumstances other Darwin Tree of Life collaborators.

## Data Availability

European Nucleotide Archive: *Dasysyrphus tricinctus* (yellow-girdled Dasysyrphus). Accession number PRJEB64114; https://identifiers.org/ena.embl/PRJEB64114 ( Wellcome Sanger Institute, 2024). The genome sequence is released openly for reuse. The *Dasysyrphus tricinctus* genome sequencing initiative is part of the Darwin Tree of Life (DToL) project. All raw sequence data and the assembly have been deposited in INSDC databases. The genome will be annotated using available RNA-Seq data and presented through the Ensembl pipeline at the European Bioinformatics Institute. Raw data and assembly accession identifiers are reported in Table 1 and Table 2.

## Author information

Members of the University of Oxford and Wytham Woods Genome Acquisition Lab are listed here: https://doi.org/10.5281/zenodo.12157525.

Members of the Darwin Tree of Life Barcoding collective are listed here: https://doi.org/10.5281/zenodo.12158331

Members of the Wellcome Sanger Institute Tree of Life Management, Samples and Laboratory team are listed here: https://doi.org/10.5281/zenodo.12162482.

Members of Wellcome Sanger Institute Scientific Operations: Sequencing Operations are listed here: https://doi.org/10.5281/zenodo.12165051.

Members of the Wellcome Sanger Institute Tree of Life Core Informatics team are listed here: https://doi.org/10.5281/zenodo.12160324.

Members of the Tree of Life Core Informatics collective are listed here: https://doi.org/10.5281/zenodo.12205391.

Members of the Darwin Tree of Life Consortium are listed here: https://doi.org/10.5281/zenodo.4783558.
