# Supplementary material for: The genome sequence of the yellow-girdled Dasysyrphus, Dasysyrphus tricinctus (Fallén, 1817)
Source: Wellcome Open Res. 2024 Oct 1;9:561. [Version 1] doi: 10.12688/wellcomeopenres.22892.1 (PMC11499735; doi:10.12688/wellcomeopenres.22892.1)
Supplement: Peer review history

# Reviewer report: Reviewer response for version 1

**DOI:** 10.21956/wellcomeopenres.25208.r103769

**Published:** 2024-10-23

**Authors:** Henrique Antoniolli, Maria Eduarda Deluca João

**Affiliations:** 1 Universidade Federal do Rio Grande do Sul (UFRGS), Porto Alegre, Brazil; 2 Department of Molecular Biology and Biotechnology, Universidade Federal do Rio Grande do Sul (Ringgold ID: 28124), Porto Alegre, State of Rio Grande do Sul, Brazil

The present study brings a highly contiguous genome assembly for the yellow-girdled *Dasysyrphus* ( *Dasysyrphus tricinctus*). Background section brings information about its general biology. The text is overall concise, well written, and describes in details the employed methodology in all steps. 

 The authors should include the L50 statistics in Table 2 for reinforcing the quality in their assembly. Overall, the manuscript is of high quality, and recommended for indexing.

Are sufficient details of methods and materials provided to allow replication by others?

Yes

Is the rationale for creating the dataset(s) clearly described?

Yes

Are the datasets clearly presented in a useable and accessible format?

Yes

Are the protocols appropriate and is the work technically sound?

Yes

Reviewer Expertise:

Genomics, evolution

We confirm that we have read this submission and believe that we have an appropriate level of expertise to confirm that it is of an acceptable scientific standard.

# Reviewer report: Reviewer response for version 1

**DOI:** 10.21956/wellcomeopenres.25208.r103767

**Published:** 2024-10-23

**Authors:** Jason Charamis

**Affiliations:** 1 Foundation for Research and Technology - Hellas, Irákleion, Greece

This is a first report of the hoverfly *Dasysyrphus tricinctus* nuclear and mitochondrial genomes. The methodology is sound and data produced of high quality. Genome assembly metrics and all evaluations performed suggest a chromosome-level assembly, despite a modest sequencing coverage (estimated at 17x). The genome assembly and all data produced in this work which will be a great genomic resource for future ecological studies involving this species.

Are sufficient details of methods and materials provided to allow replication by others?

Yes

Is the rationale for creating the dataset(s) clearly described?

Yes

Are the datasets clearly presented in a useable and accessible format?

Yes

Are the protocols appropriate and is the work technically sound?

Yes

Reviewer Expertise:

Arthropod Comparative Genomics

I confirm that I have read this submission and believe that I have an appropriate level of expertise to confirm that it is of an acceptable scientific standard.
